# Hierarchical super-structure identified by polarized light microscopy, electron microscopy and nanoindentation: Implications for the limits of biological control over the growth mode of abalone sea shells

**DOI:** 10.1186/2046-1682-5-19

**Published:** 2012-09-12

**Authors:** Andreas S Schneider, Birgit Heiland, Nicolas J Peter, Christina Guth, Eduard Arzt, Ingrid M Weiss

**Affiliations:** 1 INM - Leibniz Institute for New Materials, Campus D2 2, 66123, Saarbruecken, Germany; 2 Saarland University, Saarbruecken, 66123, Germany; 3 Universität Regensburg, Biochemie I, Regensburg, 93053, Germany

## Abstract

**Background:**

Mollusc shells are commonly investigated using high-resolution imaging techniques based on cryo-fixation. Less detailed information is available regarding the light-optical properties. Sea shells of *Haliotis pulcherina* were embedded for polishing in defined orientations in order to investigate the interface between prismatic calcite and nacreous aragonite by standard materialographic methods. A polished thin section of the interface was prepared with a defined thickness of 60 μm for quantitative birefringence analysis using polarized light and LC-PolScope microscopy. Scanning electron microscopy images were obtained for comparison. In order to study structural-mechanical relationships, nanoindentation experiments were performed.

**Results:**

Incident light microscopy revealed a super-structure in semi-transparent regions of the polished cross-section under a defined angle. This super-structure is not visible in transmitted birefringence analysis due to the blurred polarization of small nacre platelets and numerous organic interfaces. The relative orientation and homogeneity of calcite prisms was directly identified, some of them with their optical axes exactly normal to the imaging plane. Co-oriented "prism colonies" were identified by polarized light analyses. The nacreous super-structure was also visualized by secondary electron imaging under defined angles. The domains of the super-structure were interpreted to consist of crystallographically aligned platelet stacks. Nanoindentation experiments showed that mechanical properties changed with the same periodicity as the domain size.

**Conclusions:**

In this study, we have demonstrated that insights into the growth mechanisms of nacre can be obtained by conventional light-optical methods. For example, we observed super-structures formed by co-oriented nacre platelets as previously identified using X-ray Photo-electron Emission Microscopy (X-PEEM) [Gilbert et al., Journal of the American Chemical Society 2008, 130:17519–17527]. Polarized optical microscopy revealed unprecedented super-structures in the calcitic shell part. This bears, in principle, the potential for in vivo studies, which might be useful for investigating the growth modes of nacre and other shell types.

## Background

In the need for optimizing the materials performance, hierarchical construction principles became genetically manifest in the growth processes of skeleton forming animals [1] over millions of years of evolution [2-6]. The mollusk shell is one of the most ancient examples of such a hierarchical material [7-11]. Shells often consist of different crystalline polymorphs of calcium carbonate minerals such as calcite and aragonite, which are embedded in organic components [12-14]. Commonly known structures are nacre, and prismatic and crossed-lamellar shell types [15,16]. Many aspects regarding the optical and mechanical properties of mollusk shells are well understood [12,17]. The most studied shell type is nacre [18-21].

Since it is difficult to precisely classify the shell structures according to crystal sizes [22], it is more appropriate to characterize natural composites according to volume fractions of organic and inorganic phases. In the case of sea shells, the mineral part makes up more than 95 wt% of the natural composite [2,23]. The relationships between structural and mechanical properties of nacre were identified on different length scales [11,22,24]. This shell type consists of mineral tablets of about 0.5–1 μm in thickness and about 5–10 μm in diameter. Its fracture toughness is relatively high (~7 MPa√m) as compared to the inorganic compound (~0.2 MPa√m), whereas the hardness is in a similar range, ~200-300 H_v_, for both nacre and aragonite [25]. In this respect, nacre differs from other biological minerals such as corals (~0.1 MPa√m, 5–10 H_v_) and calcitic egg shells [25].

However, the growth mechanisms of the different shell structures remain speculative due to the lack of high-resolution light optical techniques, which are required to study shell formation in vivo, and the semi-transparency of adult shells. A seminal SEM study was performed by Wise [26]. He reported the sheet-like structure of the organic matrix at the nacreous growth front of the gastropod *Cittarium pica* and discussed the formation of crystal stacks in the light of earlier suggestions for their columnar growth mode in *Haliotis*[26] (and refs. therein). Wise pointed out that this is in clear contrast to the horizontal layer-by-layer growth modes for pelecypod (bivalve) nacre, e.g. of *Pinctada radiata*. The morphological study of Wise [26] could not answer the question regarding whether or not the organic matrix acts as a template for crystal nucleation by epitaxy. Weiner and Traub were using X-ray diffraction and demonstrated the perpendicular orientation of some organic molecules such as chitin fibers [27] with respect to the c-axes of the aragonite crystals for gastropods, bivalves and cephalopods. In the case of *Nautilus* the degree of misorientation of the organic matrix was in the same order of magnitude as for the a and b plane of the aragonite crystals [28]. When these authors used electron diffraction in order to gradually reduce the sampling areas down to approximately the size range of a single biological cell, they identified locally ordered aragonite patterns but, on larger scales and depending on the biological species, a mosaic organization of the organic matrix with various degrees of order [29]. This suggests a gradual, kinetic selection of faster growth rates and a small probability of nucleating a new orientation. Recent X-PEEM synchrotron experiments demonstrated a high degree of order within stacks of abalone nacre vs. a considerable degree of disorder towards the neighbouring stacks [1]. The stacks of crystalline aragonite tablets in nacre are misoriented in their c-axis with respect to each other and orientational ordering of tablet stacking occurs not abruptly but gradually over a distance of 50 μm [1,30]. Initially, the orientation of nucleation sites could be completely random, as shown e.g. in Figure 8 of [21]. It can be assumed that the orientation of the first platelet existing next to the prismatic layer is determined by the roughness of the substrate (e.g. calcite prism, spherulitic aragonite, ACC precursor granules). It is well-known that nucleation sites are located within a gel-like organic matrix [10,31] and, that certain aragonite-inducing and chitin-binding proteins may form an oriented 3-dimensional nucleation site [27,32]. Although the distribution and function of such nucleation sites have not yet been identified on the molecular level and many aspects are still under debate [33], a detailed texture analysis of calcite shells using EBSD suggests that similar mechanisms are valid for molluscs in general and also for members of other phyla such as brachiopods [34,35].

The present study was motivated by the challenge to improve quantitative light-optical investigations of mollusk shells, which would be of particular interest because they bear the potential for in vivo experiments. We performed quantitative polarization analysis using liquid crystal compensator technology (LC-PolScope AbrioTM Imaging System), introduced in 1995 by Oldenbourg [36,37]. The quality and reproducibility of LC-PolScope data depends not only on sample thickness but also on structural features [38], which are in the range of the wavelength of light and below [39,40]. Hence, the strategy used here involved sample preparation according to standard metallographical methods for producing thin sections of defined thickness and orientation [41]. In parallel to LC-PolScope analysis, a detailed light-microscopic investigation of composite interfaces using materials polarization microscopy was performed. We also characterized the mechanical properties of a polished section of the nacre/prismatic (N/P) shell transition region using nanoindentation. Due to the high lateral resolution of nano-indentation, we were able to correlate the observed structural features to mechanical function.

## Results

### Incident light microscopy

**Figure 1:**
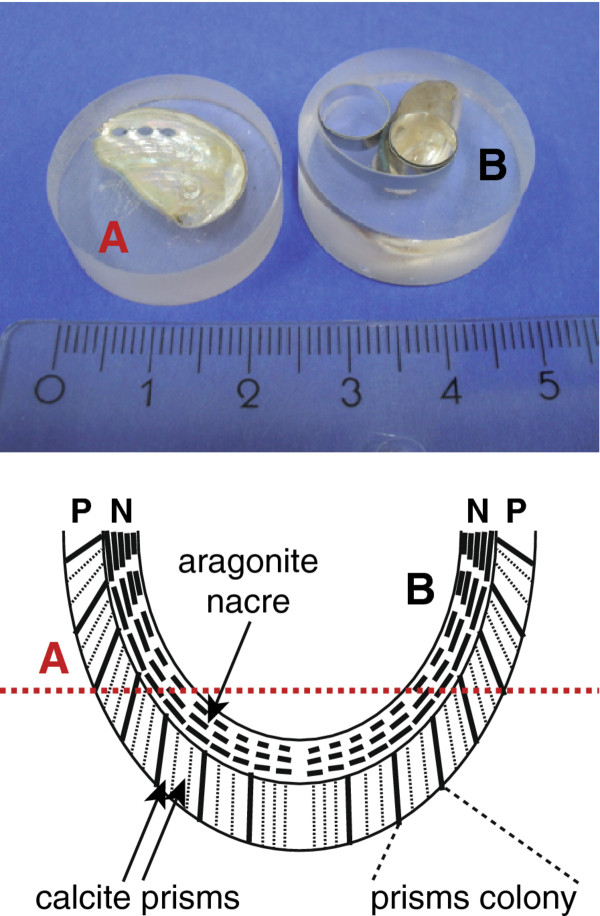
**Sample preparation.** Metallographic preparation of *Haliotis pulcherina* sea shell. Left, orientation **A**; right, orientation **B** .

The transition region between the outer prismatic calcite part and the inner aragonite (nacre) part of a *Haliotis*-type sea shell (Additional file 1: Figure S1) was prepared using standard metallographic procedures (Figure 1).

Shells were embedded in two different orientations: For orientation A, the polished section was horizontal to the coronal plane of the shell (Figure 1, left), which results in an inclination angle between the normal of each nacre platelet and the normal of the polished surface. This angle is related to the natural curvature of the *Haliotis* specimen and also depends on how much of the shell material was removed by the grinding and polishing procedure. For orientation B, the polished surface is a transverse section (Figure 1, right). In this case, the normal of each nacre platelet is perpendicular to the normal of the polished surface.

**Figure 2:**
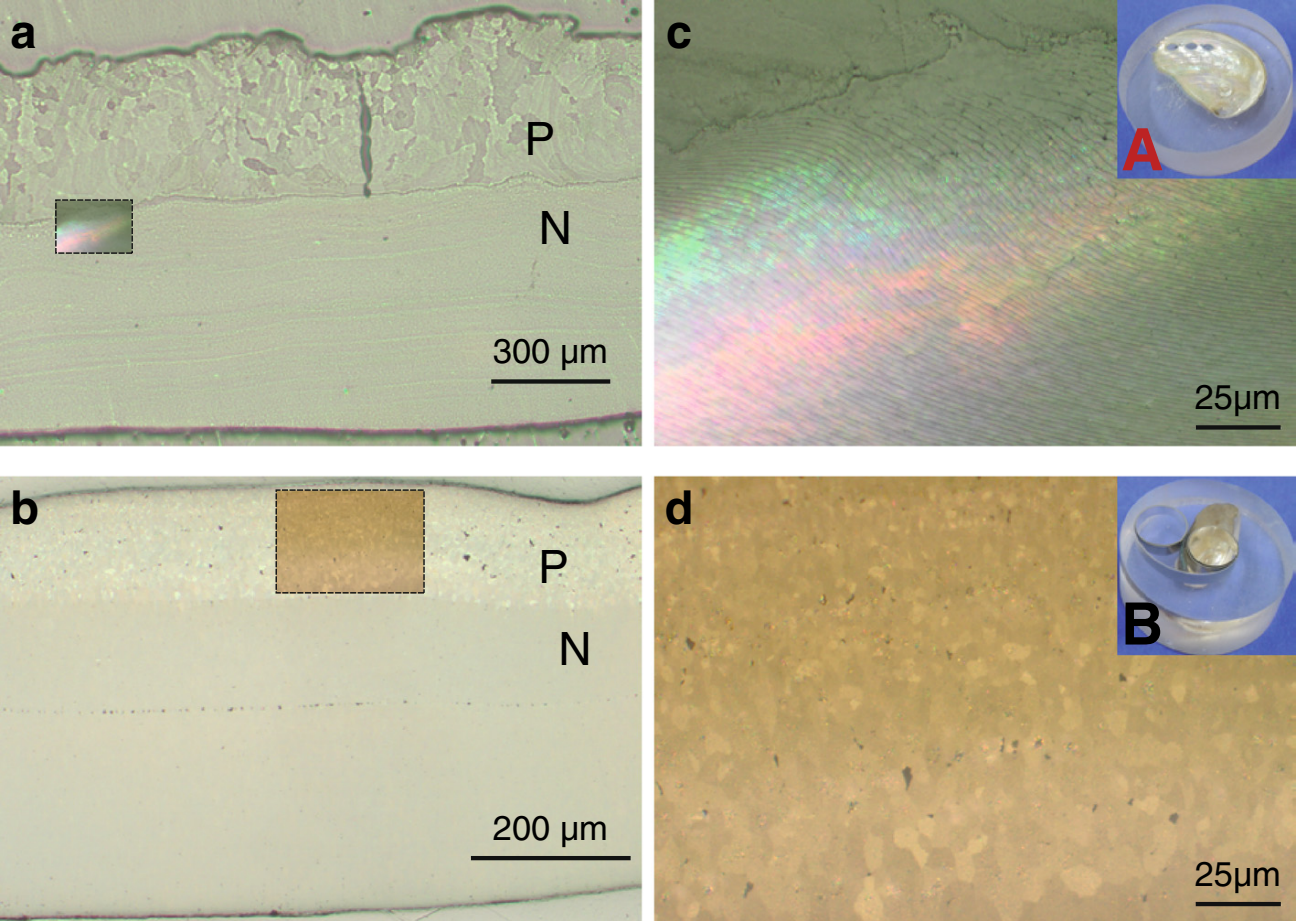
**Incident light microscopy of differently oriented shell cross-sections.** Incident light microscopy. Top, orientation **A**; bottom, orientation **B**. Hierarchical structures of nacreous and prismatic shell parts are visualized best in orientation A and B, respectively. Inserts in the images on the left side are shown at higher magnification on the right. Note that in addition to the nacre lamellae there is another level of hierarchy in the range of ~20 μm, defined here as the super-structure, which is observed in orientation A at higher magnification. P, prismatic layer; N, nacre.

The polished cross-sections were characterized using an incident light microscope. As shown in Figure 2, the sample orientation has a strong influence on the optical appearance of the two shell parts (nacre and prismatic) in the bright-field imaging mode. The lamellar structure of the nacreous part in the μm range is only visible in orientation A (Figure 2a,c), whereas the prismatic calcite part is characterized by structures in the sub-mm range which are best visualized in orientation B (Figure 2b,d). Due to the inclination angle of the nacre platelets with respect to the surface, single lamellae exceed the resolution limit of the optical microscope only in orientation A.

Besides the well-known regular nacre layers, we observed undefined colored reflexes with mosaic arrangement and dimensions covering several platelets in a stacked configuration in the incident light optical image, which cannot be corelated with surface topography. In the following, we refer to this additional contrast, which covers multiple platelets, as super-structure. (Figure 2c, Additional file 2: Figure S2).

It is known that each nacre layer reflects and transmits part of the incident white light, which causes constructive and destructive interference of the light. Due to the extinction of certain parts of the visible spectrum nacre occurs in different colors if viewed from different directions. Therefore, the iridescent properties of nacre are basically explained by Bragg's law:

(1) 2dsinθ=nλ

where *d* is the distance between reflecting planes, *θ* is the diffraction angle, *n* is the refractive index and *λ* is the wavelength of radiation. In case of nacre, where the aragonite layers are separated by organic interfaces in the size range of 10–100 nm, *d* is approximately the thickness of one aragonite layer. The diffraction angle *θ* is defined as the angle between the incident light and the reflecting nacre planes and thus depends on the orientation of the nacre platelets with respect to the polished surface.

We do not expect that the orientation as well as the thickness of the nacre platelets vary on a length scale of the super-structure. Therefore, according to Equation (1) the local color contrast can only be assigned to local changes in the refractive index, which is related to the orientation-sensitive dielectric constant of the material. The color contrast does not appear in orientation B (Figure 2b), since the reflective planes (aragonite layers) are oriented parallel to the beam and thus the Bragg condition is not fulfilled.

At this stage of the experiments it remained unclear whether the mineral crystals or the organic interfaces contribute significantly to this effect.

In order to investigate the nature of the super-structure, we chose a comparative approach using different light and electron microscopic techniques for the specimen with orientation A.

### Transmitted light microscopy

**Figure 3:**
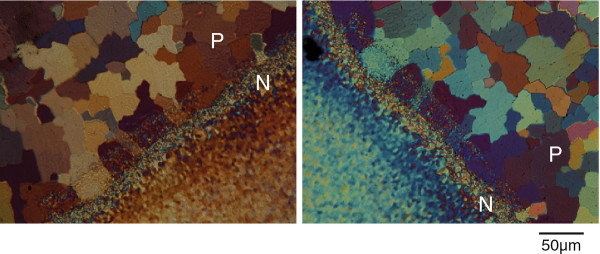
**Transmitted light microscopy of *Haliotis* shell thin section.** Polarized light microscopy in transmission of a 60 μm thin section in orientation A. The transition region nacre/prismatic is centred on the microscopy stage. The left and the right image were obtained at the same position by rotating the stage 90°. The structure of the prismatic layer (P) is clearly visible. The tapering nacre structure gradually changes as a function of distance from the prismatic layer. Note that neither the lamellar structure nor the super-structure are visible. P, prismatic layer; N, nacre.

For these experiments, a 60 μm thin section of the abalone shell in orientation A was prepared. In contrast to incident light microscopy, a clear structure of the calcite layer is visible in transmitted polarized light microsopy. It consists of domains of different sizes in the range of 5–100 μm, which appear in different colors (Figure 3). The contrast in polarized light microscopy is directly related to the 3-dimensional orientation of the unit cell of anisotropic crystals [12]. Closer inspection of the large domains reveals that these also consist of subunits in the range of 5 μm, which we interpret as the individual prisms. A rotation of the specimen by 90° did not cause an equal color change for all prisms (Figure 3a vs b). The colors of some prisms change with respect to their direct neighbours, which indicates that they have different crystallographic orientations. If the color of adjacent prisms changes in the same way, one can conclude that their relative orientations are the same. Since the prisms are inclined with respect to the surface, it remains difficult to predict the relative crystallographic orientation. Some of the prisms at the interface between calcite and aragonite remain dark (Figure 3), although no defects are present in the thin section. This suggests that these prisms have an amorphous structure or, that calcite crystals are oriented exactly with their optical axis perpendicular to the surface. The stacking of adjacent prisms with equal crystallographic orientation seems to be a structural feature of the calcitic shell part and is in the following referred to as prism colony.

Further, the previously observed lamellar structure of the nacre (Figure 2c, Additional file 2: Figure S2) is not visible in Figure 3 (N, nacreous shell part). One would expect a strong crystalline birefringence signal for aragonite for this area. However, we could not observe any structure in the sense that well-defined boundaries could be identified. This suggests that the number of interfaces in the sampled volume is too large [12]. In our case, we roughly estimate that the investigated specimen consists of ~ 60 nacre platelets interspaced by organic sheets across the transmitted section. Another explanation would be that the signals originating from crystallographically misaligned aragonite platelets are superimposed, hence causing the blurred polarization.

### LC-PolScope birefringence analysis

**Figure 4:**
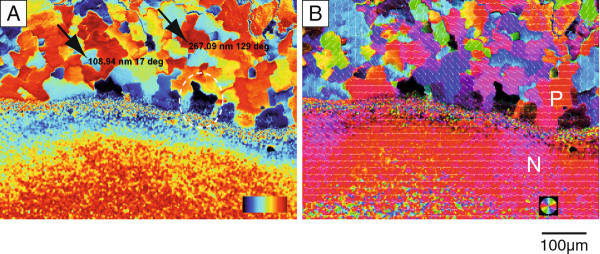
**LC-PolScope analysis of nacre/prismatic transition.** LC-PolScope ("Abrio™-Imaging") microscopy in transmission of a 60 μm section in orientation A. The birefringent retardance values (left, 0–273 nm from dark blue to dark red) and the orientation of the slow axis vector (right, red 0° / 180°, light blue 90° / 270°) are calculated for each pixel and color coded in the images. The two arrows in the left image indicate two differently oriented calcite prisms. Note that the marked light blue prism with a retardance value of ~109 nm appears orange in the orientation image (right), which corresponds to an azimutal angle of 17°. The marked red prism with a retardance value of ~267 nm appears purple in the orientation image (right), which corresponds to an azimutal angle of 129°. This analysis shows that the prisms consist of different domains, which can be distinguished according to their homogeneous crystallographic orientation. Not all of them are homogeneous. The tapering nacre layer gradually changes as a function of distance from the prismatic layer. Due to the blurred polarization signal, no structure is visible. Arrows in the left image indicate pixels with medium (ligh blue, 109 nm/17°) and high (dark red, 267 nm/129°) birefringent retardance contrast. P, prismatic layer; N, nacre.

LC-PolScope Abrio™ analysis was performed to further investigate the birefringence of calcite and aragonite structures in this 60 μm thin section of the *Haliotis* shell in transmission. Figure 4 shows the calculated birefringent retardance (Figure 4a) and orientation (Figure 4b) images of the same area as shown in Figure 3. The prism colonies in the calcite layer have almost the same morphologies as observed by conventional transmitted polarized light microscopy (Figure 3).

The orientation image (Figure 4b) indicates that the prisms cover a large range of crystallographic orientations and that adjacent prisms in a single colony have more or less similar crystallographic orientations. For differently oriented colonies the birefringent retardance values of prisms and colonies were in different ranges (e.g. 109 nm in blue areas, 267 nm in red areas; Figure 4a, arrows). For example, the prism colony with an azimutal angle of 17° (color coded in orange and indicated also by the array overlay of azimutal angles in Figure 4b) has a retardance value of 109 nm, whereas the prism colony with an azimutal angle of 129° (color coded in purple) revealed a retardance of 267 nm. In contrast to synthetic calcite [42], further comparison of Figures 4a and b shows that areas of homogeneous orientation are not necessarily related to homogeneous birefringent retardance values of the prisms. This could be due to the fact that prisms are not pure single crystals of calcite but consist of complex arrangements of organic and inorganic phases [43,44]. The interface between adjacent colonies and also adjacent prisms within the colonies appeared with non-uniform colors in both, retardance and orientation mode. This can be attributed to the organic layers surrounding the prisms [43]. Finally, the same colonies which did not change their color after 90° rotation in the conventional polarized light microscopy analysis exhibit low birefringent retardance (dark areas, Figure 4a, dotted circle) and could therefore not be identified with respect to orientation. These areas were identified by Raman imaging spectroscopy to consist of calcite (Additional file 3: Figure S3).

Although the thin section is too thick in order to quantify the birefringence in the bulk of the nacre layer, the tapering overlap region with the calcite layer can be used to obtain information regarding the orientation of individual platelets. On top of the prisms with zero retardance (Figure 4a, dotted circle), individual nacre platelets or fragments of the tapered nacreous layer were identified. Raman imaging spectroscopy confirmed that these platelets consist of aragonite (Additional file 3: Figure S3). These nacre parts appear in the blue range of retardance values. At areas where the nacreous layer is thicker and composed of several platelets stacked on the prisms, the retardance reaches the yellow-red range. This shows that the thickness of the nacreous layer significantly contributes to the observed retardance contrast. In areas where the thin section consists only of the nacreous part, a gradient of retardance values from light blue to red was detected. This phenomenon cannot directly be correlated to the structure of the sea shell and may be caused by the multiple superpositions of differently oriented nacre platelets with internal structures (Additional file 4: Figure S4 shows isolated nacre platelets investigated by LC-PolScope microscopy). Furthermore, the large fraction of interfaces contributes to form birefringence which complicates data analysis.

### Scanning electron microscopy

**Figure 5:**
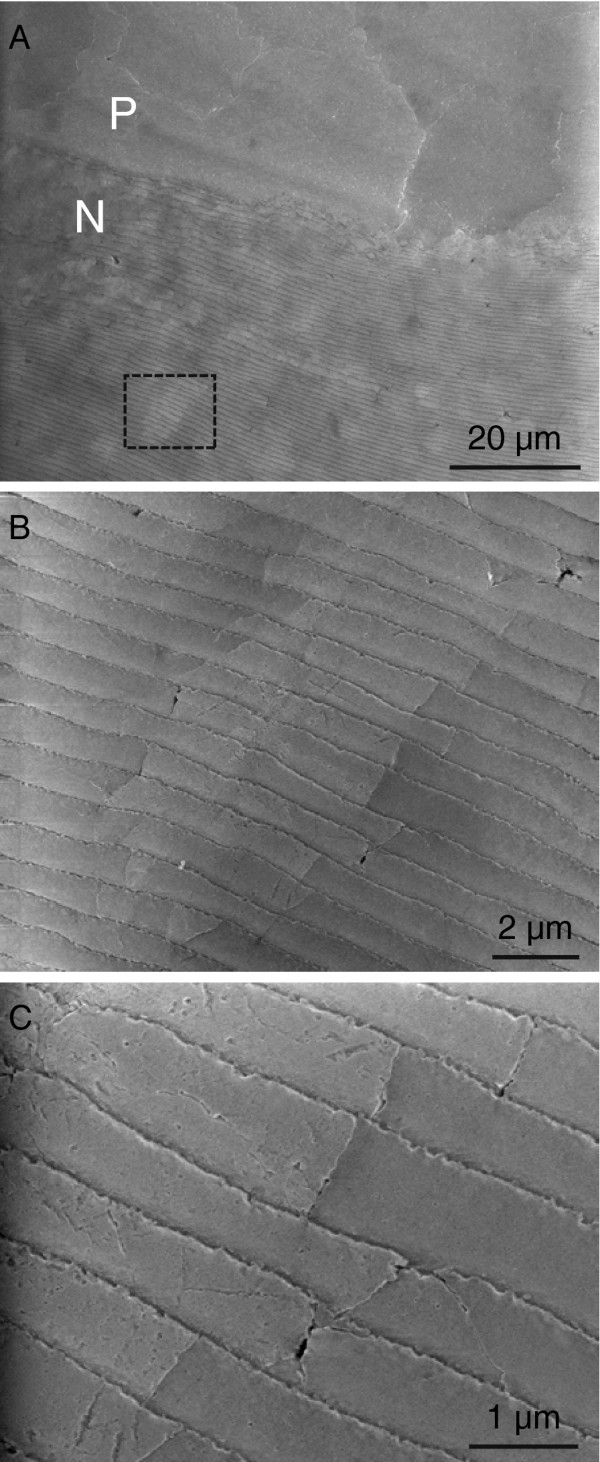
**Scanning electron microscopy image of the nacre super-structure.** Scanning electron microscopy of a 60 μm section in orientation A. Top, overview image showing the transition between prismatic and nacreous layers. Center, super-structure in the size range of 20 μm in the nacreous layer. Domains of homogeneous grey level are formed by stacks of adjacent nacre platelets. Bottom, boundary between two domains which form the super-structure.

The structure of the nacre/prisms transition region in orientation A was additionally investigated by scanning electron microscopy. As shown in Figure 5a, the nacre layer is composed of domains which differ in their grey levels. These domains encompass several stacked nacre platelets, which appear with identical grey levels. These platelet stacks are in the size range of the super-structure as observed by incident light microscopy (Figure 2c, Additional file 2: Figure S2).

At higher magnification (Figure 5b,c) it can be seen that the domain boundaries are related to the boundaries of individual nacre platelets. From the literature it is known that the c-axis orientation of the aragonite platelets is not necessarily perpendicular with respect to the plane of each individual layer, but varies [1,33,45]. Accordingly, the different grey levels of the domains might be explained by an electron channeling contrast caused by the differently oriented areas. It is well known that backscattered electrons (BE) provide information about crystallographic orientation via channeling [46]. Secondary electron (SE) images also contain this channeling contrast, though mostly superimposed by a stronger topography contrast. The LFD detector used for imaging in low vacuum collects a broader spectrum of electrons as compared to the conventional Everhard-Thorneley detector. Therefore, both SE and BE contribute to the contrasting of the image, which might explain the orientation sensitiviy of the SEM experiments with uncoated specimens. Tilting enhances the contrast as it increases the yield of backscattered electrons, reduces multiple scattering and energy loss processes and enhances channeling [46].

The fact that the super-structure was observed by two different contrasting methods, i.e. incident light microscopy and scanning electron microscopy, indicates that surface topography effects can be ruled out. The super-structure must rather be attributed to the biological growth mode [1].

### Nanoindentation

In order to investigate the implication of the super-structure on the mechanical properties of the shell, nanoindentation experiments were performed on a polished section in orientation B. This orientation was chosen as in this case the nacre platelets are parallel to the indentation axis, which makes data analysis more straight forward. The indents were performed with a maximum load of 10 mN and a distance between adjacent indents of 20 μm. Furthermore, the indents were positioned in two parallel rows such that they cover a certain part of the calcite and the nacre layer.

**Figure 6:**
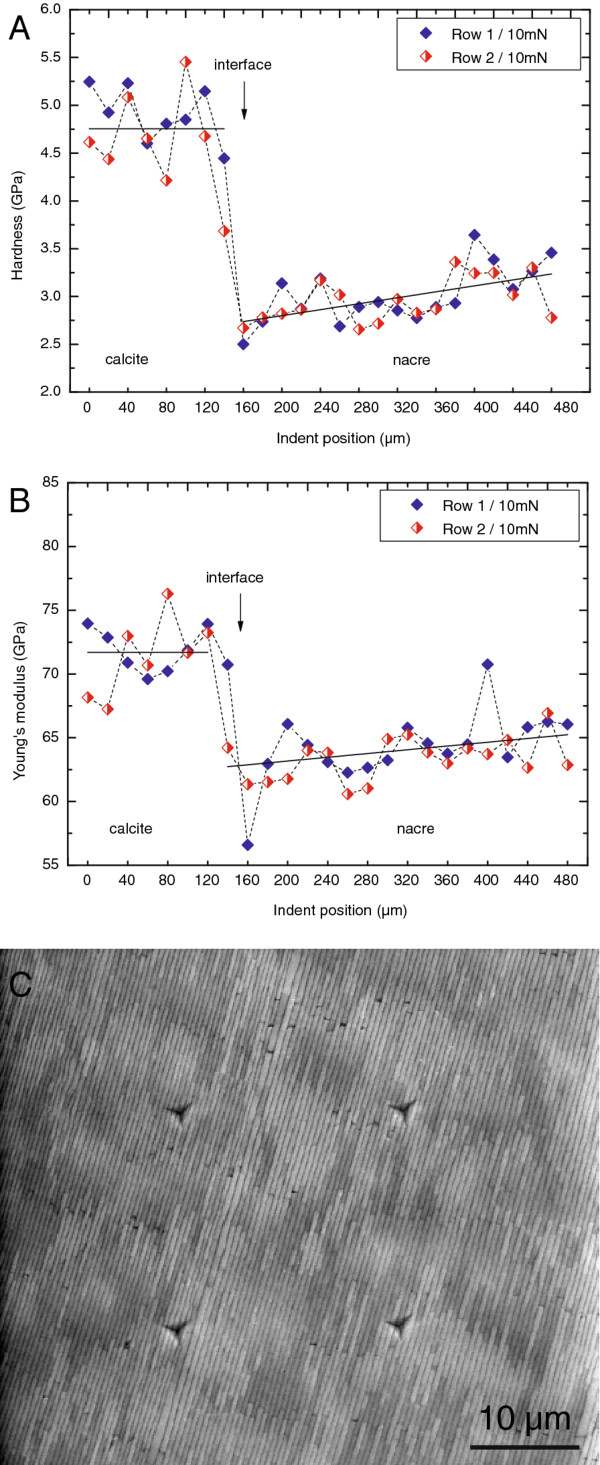
**Nanoindentation of *Haliotis* shell sectioned in orientation B.** Nanoindentation experiments performed on a polished section in orientation B. Hardness (**A**) and Young's modulus (**B**) are shown as a function of indentation position. **C**, Scanning electron microscopy image of the cross section after the experiments. The positions of some indents with respect to the 20 μm super-structure are exposed. Indents were 20 μm apart from each other and do not exactly match with the periodicity of the super-structure. Note that the super-structure in this image was visualized at a tilt angle of 20°.

Figures 6a and b show the mechanical properties of the sea shell as a function of indent position. For both rows the first indents were placed in the calcite layer and all other indents were positioned in the nacreous part. Hardness and Young’s modulus vary significantly over the investigated area, especially across the interface. For the calcite prisms, hardness values of about 4.2 to 5.2 GPa and Young’s moduli of approximately 72 ± 2 GPa were found. For the nacre layer, lower values of 2.5 to 3.7 GPa and about 65 ± 2 GPa were found. In addition, it was observed that both hardness and Young's modulus slightly increase in the nacre layer from the interface calcite – aragonite to the inner surface of the shell.

Another observation in Figure 6 is the systematic variation in hardness and Young’s modulus for calcite and aragonite. As the distance between adjacent indents is similar to the characteristic size of the previously observed super-structure, this variation could reflect the different properties of individual domains of the super-structure. This is demonstrated in Figure 6c, which shows an SEM image of residual indents in the polished section. The image was obtained under a defined tilt angle of 20° in order to visualize the super-structure in the SEM even for the sample in orientation B. Note that indents were randomly distributed over areas (platelet stacks) with different grey levels.

## Discussion

**Figure 7:**
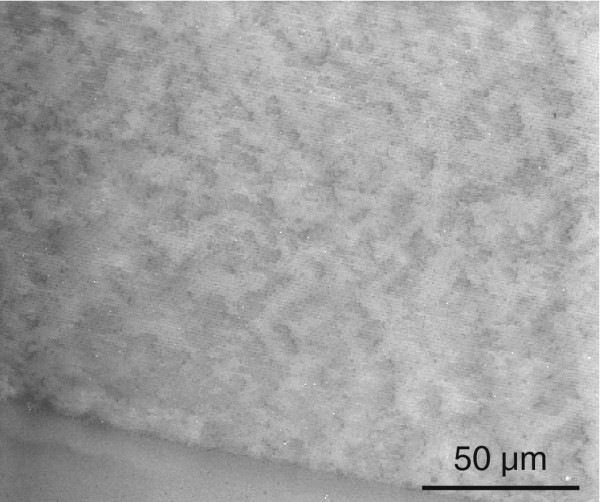
**Scanning electron microscopy image of nacre super-structures.** Scanning electron microscope image of a *Mytilus galloprovincialis* sea shell. Domains of homogeneous grey level are formed by stacks of adjacent nacre platelets.

Here we report the investigation of carefully prepared polished sections and a semi-transparent thin-section of the nacre-prismatic transition region of *Haliotis pulcherina* shells. The combination of conventional incident light optical microscopy, quantitative polarized light microscopy (LC-PolScope AbrioTM system) in transmission, VP-SEM and nanoindentation enabled the detection of super-structures in both, nacre and prismatic shell regions. In *Haliotis rufescens* nacre, a similar structure was previously identified by X-PEEM [1]. Actually, we applied the same light microscopy and VP-SEM methods to the nacreous layer of the bivalve *Mytilus* and observed also a super-structure comparable to the one in *Haliotis pulcherina* (Figure 7). Observing the same feature in both, gastropods and bivalves, with completely different growth modes in nacre [31], raises the question whether this structural feature is evolutionary advantageous, or whether it is the result of mineralization processes. For example, the experimental observations made by Gilbert et al. [1] agreed well with kinetic growth models [30]. On the other hand, for other controlled biomineralization systems such as bone hydroxyapatite and sponge silica, the formation of distinct super-structures are mechanically advantageous [47]. Accordingly, super-structures can be an evolutionary advantage and therefore genetically conserved "on purpose" in mollusc species in general, regardless of their particular growth modes.

The biological control mechanisms are well understood on the level of a single nacre platelet [31]. However, mechanisms explaining the overall structure of shells are still not clear. With respect to the super-structure observed here, we suggest the following scenario: The formation of the very first nacre layer starts at the irregular growth front with the deposition of nucleation seeds [21], which are most likely specific proteins secreted by the tissue. Hence, the nacre platelets originating from these seeds will be randomly oriented with respect to their aragonite c-axis. Since the growth kinetics is directly related to the crystallographic orientation of the growing crystal, it is more likely that platelets with a prefered orientation grow on the expense of less well oriented platelets and thus occupy more space in a given time. Once another layer is deposited and new seed crystals start to grow, those growing on top of the "well oriented" platelets, which occupy already a much larger space than the less well oriented ones, have a higher probability to grow faster and with the prefered orientation. Although mineral bridges could contribute to transfer crystal orientation on the level of adjacent nacre platelets within one stack of platelets, they can not account for the super-structure, which is composed of differently oriented platelet stacks. On the level of competing stacks of platelets, the orientation, concentration and arrangement of nucleation seeds would be predetermined by the organic matrix.

The same may apply for prisms, which finally appear as prism colonies with aligned orientations of adjacent crystals.

In order to see whether the super-structures affect the mechanical properties, which would be an evolutionary advantage [47,48], indentation experiments were performed on the size scale of the super-structure.

Overall, hardness and Young's modulus values found for *Haliotis pulcherina* nacre are very similar to those recently reported [49-52]. Using microindentation, for example Fleischli et al. [52] obtained a hardness of 3.9 GPa for *Haliotis rufescencs*. The Young's modulus in their study was only measured by nanoindentation. At low penetration depths, an average value of 72 GPa was detected. However, it was realized that with increasing penetration depth, the Young's modulus droped significantly. For example, at a penetration depth of 160 nm they found a modulus of 65 GPa, which is very similar to the results of the current study. The strong influence of the penetration depth in [52] was explained by the indentation size effect and an increasing contribution of the soft organic matrix with increasing penetration depth. Note that for geological aragonite without any polymer inclusions the nanohardness is in a range of 6.2 ± 0.3 GPa and Young's modulus is in the order of 102.8 ± 2.4 GPa [53].

The influence of the organic matrix may also contribute to the different mechanical properties of the calcite and aragonite layer. Both layers consist of mineralized calcium carbonate and a small fraction of biopolymer. However, in the regular brick and mortar structure of the nacre, each calcium carbonate platelet is surrounded by a soft matrix on a length scale, which is sensed by nanoindentation. Therefore, in nacre the organic phase has to carry the same load as the hard particles, which causes the lower hardness and Young's values compared to calcite. The influence of the organic layer can be estimated by simple composite models [54] or, more realistically, by the "shear lag model" [55].

The gradual change of the mechanical properties in the nacre layer is likely related to changes of structural features along the cross-section of the sample. In particular, Pokroy et al. [56] have shown that the thickness of the nacre platelets increases from the interface calcite/aragonite to the inner surface of the shell. Similar changes in structural features were observed by Nudelman et al. [18] at the growth front of nacre. Therefore, it can be assumed that the contribution of the softer organic matrix to the mechanical properties decreases as the positions of the indents approach the inner surface. This explains the higher hardness and Young’s modulus values for indents performed closer to the inner surface of the shell.

The systematic variation in hardness and Young’s modulus for calcite and aragonite, might partially be explained by topography effects, or locally different contributions of the soft organic matrix. However, also the crystallographic orientation of the indented structural features might contribute to this effect. Our experiments confirm the original observations made by Schmidt [12], who showed that individual prisms in the calcite layer have different crystallographic orientations. Therefore, the scatter in the Young’s modulus can be explained by the fact, that indents were performed in prisms with a soft or hard crystallographic direction [57,58].

For the nacre layer, where the size of the structural features is smaller than the indented area, the influence of the crystallographic orientation is less obvious. Normally it would be expected that the influence of the crystallographic orientation of the platelets is averaged out, if several platelets are indented at the same time. However, the super-structure observed in this work and the work by Gilbert et al. [1] indicate that groups of adjacent platelets have an equal crystallographic orientation. Thus the scatter in the indentation data likely reflects the different orientations of these tablet stacks and is an example of where the observed super-structure influences the mechanical properties of sea shell.

All methods applied in this study enable a fast and easy screening of different types of mollusc shells. This is important to identify different growth modes as a function of cell physiology and biochemistry. Here we successfully demonstrated using combined methods that both, nacre and prismatic shell parts consists of characteristic super-structures at the nacre-prismatic interface. We also demonstrate that the super-structures correlate with distinct mechanical properties. These results allow us to draw conclusions regarding the limits of biological control.

## Conclusions

Extensive studies targeted the crystallographic orientation of aragonite platelets in the nacreous layer and calcite prisms in the prismatic layer with respect to the overall shape and function of mollusc shells [12,17]. However, hierarchical super-structures have to the best of the authors knowledge not yet reported to be obtained with conventional light optical and SEM techniques, only with much more sophisticated methods such as synchrotron X-PEEM and TEM. Here, we identified an additional contrast in the incident light microscopy and interpret this as additional nacre and prismatic super-structures. Both super-structures encompass several subunits of the structure such as nacre platelets and calcite prisms. Our observations have major implications for the growth mode, the limits of biological control, and the potential consequences for shell mechanics in various species and developmental stages. This bears an enormous potential for in vivo studies using conventional optical techniques, which might be useful to shed new light on the growth modes of nacre, the prismatic interface and other shell types in real time.

## Methods

### Sample preparation

*Haliotis pulcherina* shells of 10–20 mm diameter were obtained commercially (U.S. Shell Inc., Los Fresnos, Texas, U.S.A.). We followed established procedures for sample processing [59-61]. In brief, shell pieces were mounted in two different orientations at room temperature in epoxy resin (Epofix, Struers, Willich, Germany) using a vacuum impregnation device (Cast N’Vac-1000, Buehler, Düsseldorf, Germany).

Flat cross-sections were obtained by SiC paper grinding at grain sizes of 46, 26, 15, and 8 μm (Buehler, Düsseldorf, Germany), subsequent polishing with “Dur” cloths and abrasives of 6 μm and 3 μm, and “Mol” cloths and abrasives of 1 μm (Struers, Willich, Germany), respectively. Specimens were endpolished using “NAP” cloths and OP-S suspension (Struers), rinsed extensively with water and ethanol, and air dried. The quality of the cross-sections and the cleanliness of the surfaces were confirmed in reflected bright field mode.

Thin sections were obtained by mounting the polished flat cross-section onto a glass slide using the epoxy resin (Epofix, Struers, Willich, Germany) and abrasing the epoxy resin from the top towards the shell to a final specimen thickness of 60 μm. The final thin section was again endpolished.

### Incident light microscopy

Reflected bright field mode microscopy was performed using an inverted research metallurgical microscope PMG3 (Olympus, Hamburg, Germany) equipped with NeoSPlan 10 NIC 0.25 ∞/- f = 180, NeoSPlan 20 NIC 0.4 ∞/0 f = 180 and NeoSPlan 50 NIC 0.7 ∞/0 f = 180 objectives. Images were recorded at 2080 × 1544 pixel resolution with the Olympus Digital Color Camera UC30 and "analySIS start" (Olympus Soft Imaging Solutions GmbH; http://www.olympus-sis.com; Münster, Germany).

This microscope with the same objectives, and with crossed polarizers and Nicol's prism was used for quality control of polished surfaces in DIC (differential interference contrast) mode.

### Transmitted light microscopy

Transmitted light microscopy was performed using a wide-field polarization microscope Orthoplan-Pol (Leitz, Wetzlar, Germany) equipped with NPL FLUOTAR 16×/0.45 P and NPL FLUOTAR 25×/0.55 P objectives. Images were recorded with an external 7.5 MPixel Live View Digital SLR Camera (E-330 ZUIKO DIGITAL 14–45 mm 1:3.5–5.6 ; Olympus Imaging Corp., http://www.olympus-europa.com) with a C-mount adaptor. The camera settings for taking the electronic images were optimized according to the best match with manual adjustments for visual analysis.

### LC-PolScope birefringence analysis

An LC-PolScope image processing system (CRI, LOT-Oriel, Darmstadt, Germany) was used for quantitative birefringence analysis at 546 nm using the standard Abrio™ filter. The LC-PolScope camera was mounted on a Zeiss inverted microscope (Zeiss Observer Z1, Göttingen, Germany) equipped with A-Plan 10×/0.25Ph1, LD Plan Neofluar 20×/0.4 Korr Ph2, LD Plan Neofluar 40×/0.6Korr Ph2, and Plan Achromat 63×/1.40 Oil DIC objectives. All images were obtained according to the manufacturers instructions. Background images were taken from a selected area of the transparent glas slide without the biological sample. The Abrio™ image processing software (CRI/LOT-Oriel; [62,63]) was used for quantitative data evaluation per pixel with respect to both, the retardance mode and the orientation of the slow optical axis (azimuth) mode.

### Raman imaging microscopy

The mineralogy of the specimens was investigated at room temperature in air using a confocal LabRAM ARAMIS Vis Raman Imaging Spectrometer (Horiba Jobin Yvon, Bensheim, Germany), equipped with 50×/0.75 WD = 0.37 mm air objective Olympus MPlan (Olympus, Germany), and a diode laser 785 nm, 80 mW (Pilot P500, Sacher Lasertechnik Group, Marburg, Germany). Spectra represent the average of 2 measurements with an exposure time of 4 sec.

### Scanning electron microscopy (VP-SEM)

Specimens were investigated without any coating in low-vacuum mode at 100 Pa using a FEI Quanta VP-SEM (FEI, Eindhoven, Netherlands), equipped with a secondary electon detector, ETD Everhard-Thornley-Detector and a LFD Large-Field-Detector for low vacuum imaging. Images were obtained at 10 keV and 15 keV (working distance 7 and 10 mm) at room temperature.

### Mechanical testing, nanoindentation

Nanoindentation experiments were performed on polished sections of shell specimens using a Hysitron Triboindenter TI 950 equipped with a standard transducer and a Berkovich tip. In total, two rows of 25 indents with peak loads of 10 mN were applied along the cross-section of the shell. The distance between two adjacent indents was 20 μm and tests were run in open-loop mode. For the extraction of hardness and Young’s modulus from the data, the unloading segment of the curves was analyzed by the Oliver and Pharr method [64].

### Isolation of single nacre platelets

Abalone shells (*Haliotis pulcherina*) were rinsed with tap water and dried with clean tissue. The outer prismatic calcite shell layers were mechanically and chemically removed using 0.6 M HCl. The remaining acid was completely removed with pure water and dried in air. Nacreous shell parts were treated with sodium hypochlorite solution (12% active chlorine) for 24 hours and the platelet fraction was recovered from the turbid supernatant by 2 min. centrifugation at 3000 rpm (Rotanta 460 RS with swing out rotor, Hettich, Germany) at room temperature. Subsequent washing steps of the solid phase were performed 20× with pure water until any chlorine vapour was completely removed.

## Competing interests

The authors declare that they have no competing interests.

## Authors’ contributions

BH carried out the metallographic specimen preparation and light optical analysis. ASS and NJP carried out the electron microscopy experiments and mechanical testing. CG participated in the specimen preparation and performed spectroscopy measurements. BH and EA participated in the interpretation of data. ASS and IMW conceived of the study, and participated in its design and coordination, and interpretation of data. ASS, BH, EA and IMW drafted the manuscript. All authors read and approved the final manuscript.

## Supplementary Material

Additional file 1 **Fractured shell at the nacre/prismatic transition Left image, Environmental scanning electron microscopy image of a fractured *Haliotis pulcherina* sea shell.** The sharp transition from prismatic calcite (top layer) to aragonite nacre (bottom layer) is formed by differentiated shell forming regions of the mantle epithelial tissue. The biological regulation of such sharp transitions between different shell structures is still enigmatic. Right image, scanning electron microscopy image of the nacreous part of a fractured *Haliotis pulcherina* sea shell. The regular stacking of single nacre platelets is visualized, whereas a super-structure in the size range of up to 20 single platelets in vertical direction is not observed here. Click here for file

Additional file 2 **Incident light microscopy of nacre shell part.** Incident light microscopy of polished shell cross-section in orientation A (compare also Figure 2C). In addition to the nacre lamellae there is another level of hierarchy in the range of ~20 μm, defined here as the super-structure, which is observed in orientation A at higher magnification. Click here for file

Additional file 3 **Raman imaging microscopy of *Haliotis* shell thin section.** The transition region contains overlapping calcite and aragonite phases, which can be clearly identified by Raman imaging spectroscopy. Raman spectra of two different calcite regions are shown. Prisms with high birefringent retardance values were identified as calcite (red spectrum). The prisms which appeared black with zero retardance in LC-PolScope microscopy are clearly identified as calcite (purple / dark blue spectrum). Aragonite spectra were obtained in tapering nacre shell parts close to the transition region, partly with some calcite finger-print (blue-green, light blue spectra). Spectra were not background subtracted with respect to the epoxy resin (green). Click here for file

Additional file 4: **Birefringent retardance of isolated nacre platelets.** LC-PolScope orientation image of isolated nacre tablets from *Haliotis pulcherina* sea shell. In addition to single nacre platelets (arrow), clusters of platelets attached to one another in a fragment of a single nacre layer are also visible. The overall birefringence in the c-axis direction of each platelet is weak, whereas the form birefringence at the boundary between mineral and water gives a bright contrast. This is demonstrated by the fact that confluent platelets show internal boundaries between single platelets with only weak retardance values. Dot-like structures are also visible, which have similar orientations within the imaging plane (a- and b-axis of the aragonite crystals) in certain domains of each platelet. The dot-like patterns could be internal or surface structures. If they are internal structures, they could be organic matrix shielded by the mineral phase or, replica voids as a result of sodium hypochlorite treatment, which was used to solubilize the interlamellar organic matrix. They could as well be the result of surface topography. Click here for file

## References

[B1] Gilbert PUPA Metzler RA Zhou D Scholl A Doran A Young A Kunz M Tamura N Coppersmith SN Gradual ordering in red abalone nacre J Am Chem Soc 2008 130 17519 17527 10.1021/ja8065495 19049281

[B2] Currey JD Taylor JD The mechanical behaviour of some molluscan hard tissues J Zool 1974 173 395 406

[B3] Lowenstam HA Weiner S On Biomineralization 1989 New York: Oxford Univ. Press

[B4] Simkiss K Wilbur KM Biomineralization: Cell Biology and Mineral Deposition 1989 CA (U.S.A.): Academic, San Diego

[B5] Dunlop JWC Fratzl P Biological composites Annu Rev Mater Res 2010 40 1 24 10.1146/annurev-matsci-070909-104421

[B6] Wegst UGK Ashby MF The mechanical efficiency of natural materials Philos Mag 2004 84 2167 2181 10.1080/14786430410001680935

[B7] Mann S Biomineralization. Principles and concepts in bioinorganic materials chemistry 2001 New York: Oxford Univ. Press

[B8] Meldrum FC Cölfen H Controlling mineral morphologies and structures in biological and synthetic systems Chem Rev 2008 108 4332 4432 10.1021/cr8002856 19006397

[B9] Weiner S Mahamid J Politi Y Ma Y Addadi L Overview of the amorphous precursor phase strategy in biomineralization Front Mater Sci Chin 2009 3 104 108 10.1007/s11706-009-0036-x

[B10] Addadi L Joester D Nudelman F Weiner S Mollusk shell formation: a source of new concepts for understanding biomineralization processes Chem - A Eur J 2006 12 980 987 10.1002/chem.200500980 16315200

[B11] Barthelat F Nacre from mollusk shells: a model for high-performance structural materials Bioinspir Biomim 2010 5 035001 10.1088/1748-3182/5/3/035001 20729573

[B12] Schmidt WJ Die Bausteine des Tierkörpers in Polarisiertem Lichte 1924 Bonn: Cohen

[B13] Towe KM Hamilton GH Ultrastructure and inferred calcification of the mature and developing nacre in bivalve mollusks Calcif Tissue Int 1967 1 306 318 10.1007/BF02008102 5654989

[B14] Rousseau M Lopez E Stempflé P Brendlé M Franke L Guette A Naslain R Bourrat X Multiscale structure of sheet nacre Biomaterials 2005 26 6254 6262 10.1016/j.biomaterials.2005.03.028 15907339

[B15] Bøggild OB The shell structure of the mollusks K Dan Vidensk Selsk Skr Naturvidensk Math Afd 1930 9 233 326

[B16] Taylor JD Kennedy WJ Hall A The shell structure and mineralogy of the bivalvia Bull Br Mus Nat Hist (Zool) 1973 22 253 294

[B17] Currey JD Mechanical Properties of Mollusc Shell The mechanical properties of biological materials 1980 U.K: Cambridge Univ. Press, Cambridge

[B18] Nudelman F Shimoni E Klein E Rousseau M Bourrat X Lopez E Addadi L Weiner S Forming nacreous layer of the shells of the bivalves Atrina rigida and Pinctada margaritifera: An environmental- and cryo-scanning electron microscopy study J Struct Biol 2008 162 290 300 10.1016/j.jsb.2008.01.008 18328730

[B19] Jäger C Cölfen H Fine structure of nacre revealed by solid state 13C and 1H NMR CrystEngComm 2007 9 1237 1244 10.1039/b708600h

[B20] Mukai H Saruwatari K Nagasawa H Kogure T Aragonite twinning in gastropod nacre J Cryst Growth 2010 312 3014 3019 10.1016/j.jcrysgro.2010.07.002

[B21] Zaremba CM Belcher AM Fritz M Li Y Mann S Hansma PK Morse DE Speck JS Stucky GD Critical transitions in the biofabrication of abalone shells and flat pearls Chem Mater 1996 8 679 690 10.1021/cm9503285

[B22] Barthelat F Li CM Comi C Espinosa HD Mechanical properties of nacre constituents and their impact on mechanical performance J Mater Res 2006 21 1977 1986 10.1557/jmr.2006.0239

[B23] Sarikaya M Gunnison KE Yasrebi M Aksay IA Mechanical property-microstructural relationships in abalone shell MRS Proc 1989 174 109

[B24] Meyers MA Lin AYM Chen PY Muyco J Mechanical strength of abalone nacre: Role of the soft organic layer J Mech Behav Biomed Mater 2008 1 76 85 10.1016/j.jmbbm.2007.03.001 19627773

[B25] Ashby MF Design G The CES EduPack database of natural and man-made materials Granta Material Inspiration - Bioengineering 2008 U.K: Granta Design, Cambridge

[B26] Wise SW Microarchitecture and deposition of gastropod nacre Science 1970 167 1486 1488 10.1126/science.167.3924.1486 17750343

[B27] Levi-Kalisman Y Falini G Addadi L Weiner S Structure of the nacreous organic matrix of a bivalve mollusk shell examined in the hydrated state using Cryo-TEM J Struct Biol 2001 135 8 17 10.1006/jsbi.2001.4372 11562161

[B28] Weiner S Traub W X-ray diffraction study of the insoluble organic matrix of mollusk shells FEBS Lett 1980 111 311 316 10.1016/0014-5793(80)80817-9

[B29] Weiner S Talmon Y Traub W Electron diffraction of mollusc shell organic matrices and their relationship to the mineral phase Int J Biol Macromol 1983 5 325 328 10.1016/0141-8130(83)90055-7

[B30] Coppersmith SN Gilbert PUPA Metzler RA Theoretical characterization of a model of aragonite crystal orientation in red abalone nacre J Phys A Math Theor 2009 42 125101 10.1088/1751-8113/42/12/125101

[B31] Nudelman F Gotliv BA Addadi L Weiner S Mollusk shell formation: Mapping the distribution of organic matrix components underlying a single aragonitic tablet in nacre J Struct Biol 2006 153 176 187 10.1016/j.jsb.2005.09.009 16413789

[B32] Suzuki M Saruwatari K Kogure T Yamamoto Y Nishimura T Kato T Nagasawa H An acidic matrix protein, Pif, is a key macromolecule for nacre formation Science 2009 325 1388 1390 10.1126/science.1173793 19679771

[B33] Checa AG Cartwright JHE Willinger M-G Mineral bridges in nacre J Struct Biol 2011 176 330 339 10.1016/j.jsb.2011.09.011 21982842

[B34] Hahn S Rodolfo-Metalpa R Griesshaber E Schmahl WW Buhl D Hall-Spencer JM Baggini C Fehr KT Immenhauser A Marine bivalve geochemistry and shell ultrastructure from modern low pH environments BGD Biogeosci Discuss 2011 8 10351

[B35] Goetz AJ Steinmetz DR Griesshaber E Zaefferer S Raabe D Kelm K Irsen S Sehrbrock A Schmahl WW Interdigitating biocalcite dendrites form a 3-D jigsaw structure in brachiopod shells Acta Biomater 2011 7 2237 2243 10.1016/j.actbio.2011.01.035 21295163

[B36] Oldenbourg R Goldman RD, Spector DL Polarization microscopy with the LC-PolScope Live Cell Imaging: A Laboratory Manual 2004 New York: Cold Spring Harbor Laboratory Press 205 237

[B37] Oldenbourg R Mei G New polarized light microscope with precision universal compensator J Microsc 1995 180 140 147 10.1111/j.1365-2818.1995.tb03669.x 8537959

[B38] Eder M Lütz-Meindl U Weiss IM Non-invasive LC-PolScope imaging of biominerals and cell wall anisotropy changes Protoplasma 2010 246 49 64 10.1007/s00709-010-0124-x 20232089

[B39] Alberts B Johnson A Lewis J Raff M Roberts K Walter P Molecular Biology of the Cell 2007 5 U.S.A: Garland Science, New York

[B40] Nelson DL Cox MM Lehninger Principles of Biochemistry 2005 New York: W.H. Freeman & Co

[B41] Heiland B Schneider AS Arzt E Weiss IM Kneissl A, Frankfurt CH Skalenübergreifende Strukturanalyse an Anschliffen und Dünnschliffen von Seeohr-Schalen Fortschritte in der Metallographie - Vortragstexte der 13 Internationalen Metallographie-Tagung, 29 September - 01 Oktober 2010 in Leoben. Volume 42 2010 Germany: Werkstoff-Informationsgesellschaft mbH 121 126 Petzow G (Series Editor): Sonderbände der Praktischen Metallographie.

[B42] Oldenbourg R Polarized light field microscopy: an analytical method using a microlens array to simultaneously capture both conoscopic and orthoscopic views of birefringent objects J Microsc 2008 231 419 432 10.1111/j.1365-2818.2008.02053.x 18754996 PMC2535855

[B43] Nudelman F Chen HH Goldberg HA Weiner S Addadi L Spiers memorial lecture. Lessons from biomineralization: Comparing the growth strategies of mollusc shell prismatic and nacreous layers in Atrina rigida Faraday Discuss 2007 136 9 25 discussion 107–123 17955800 10.1039/b704418f

[B44] Gilbert PUPA Young A Coppersmith SN Measurement of c-axis angular orientation in calcite (CaCO3) nanocrystals using X-ray absorption spectroscopy Proc Natl Acad Sci USA 2011 108 11350 11355 10.1073/pnas.1107917108 21693647 PMC3136314

[B45] Olson IC Kozdon R Valley JW Gilbert PUPA Mollusk shell nacre ultrastructure correlates with environmental temperature and pressure J Am Chem Soc 2012 134 7351 7358 10.1021/ja210808s 22313180

[B46] Reimer L Scanning Electron Microscopy: Physics of Image Formation and Microanalysis 1998 2 Heidelberg: Springer, Berlin

[B47] Fratzl P Gupta HS Fischer FD Kolednik O Hindered crack propagation in materials with periodically varying Young's modulus - Lessons from biological materials Adv Mater 2007 19 2657 10.1002/adma.200602394

[B48] Perez-Huerta A Cusack M Zhu W Assessment of crystallographic influence on material properties of calcite brachiopods Miner Mag 2008 72 563 568 10.1180/minmag.2008.072.2.563

[B49] Sun J Bhushan B Hierarchical structure and mechanical properties of nacre: A review RSC Adv 2012 2 7617 7632 10.1039/c2ra20218b

[B50] Li X Chang W-C Chao YJ Wang R Chang M Nanoscale structural and mechanical characterization of a natural nanocomposite material: the shell of red abalone Nano Lett 2004 4 613 617 10.1021/nl049962k

[B51] Katti KS Mohanty B Katti DR Nanomechanical properties of nacre J Mater Res 2006 21 1237 1242 10.1557/jmr.2006.0147

[B52] Fleischli FD Dietiker M Borgia C Spolenak R The influence of internal length scales on mechanical properties in natural nanocomposites: a comparative study on inner layers of seashells Acta Biomater 2008 4 1694 1706 10.1016/j.actbio.2008.05.029 18617447

[B53] Kearney C Zhao Z Bruet BJF Radovitzky R Boyce MC Ortiz C Nanoscale anisotropic plastic deformation in single crystal aragonite Phys Rev Lett 2006 96 255505 16907321 10.1103/PhysRevLett.96.255505

[B54] Mayer G Sarikaya M Rigid biological composite materials: Structural examples for biomimetic design Exp Mech 2002 42 395 403 10.1007/BF02412144

[B55] Jackson AP Vincent JFV Turner RM The mechanical design of nacre Proc R Soc Lond B Biol Sci 1988 234 415 10.1098/rspb.1988.0056

[B56] Pokroy B Demensky V Zolotoyabko E Nacre in mollusk shells as a multilayered structure with strain gradient Adv Funct Mater 2009 19 1054 1059 10.1002/adfm.200801201

[B57] Bhimasenachar J Elastic constants of calcite and sodium nitrate Proc Math Sci 1945 22 199 208

[B58] Dandekar D Variation in the elastic constants of calcite with temperature J Appl Phys 1968 39 3694 10.1063/1.1656842

[B59] Petzow G Metallographisches, keramographisches und plastographisches Ätzen 2006 Stuttgart: Borntraeger, Berlin

[B60] Schumann H Metallographie 1990 Stuttgart: Deutscher Verlag für Grundstoffindustrie

[B61] Telle R Petzow G Cahn RW, Haasen P, Kramer EJ Light Microscopy Materials Science and Technology 1992 Weinheim: Verlag Chemie 357 422

[B62] Shribak M Oldenbourg R Techniques for fast and sensitive measurements of two-dimensional birefringence distributions Appl Opt 2003 42 3009 3017 10.1364/AO.42.003009 12790452

[B63] Oldenbourg R Analysis of microtubule dynamics by polarized light Methods Mol Med 2007 137 111 123 10.1007/978-1-59745-442-1_8 18085225 PMC2276855

[B64] Oliver WC Pharr GM Improved technique for determining hardness and elastic modulus using load and displacement sensing indentation experiments J Mater Res 1992 7 1564 1580 10.1557/JMR.1992.1564

