# Supplementary figures and images for: Hierarchical super-structure identified by polarized light microscopy, electron microscopy and nanoindentation: Implications for the limits of biological control over the growth mode of abalone sea shells
Source: BMC Biophys. 2012 Sep 12;5:19. doi: 10.1186/2046-1682-5-19 (PMC3507795; doi:10.1186/2046-1682-5-19)

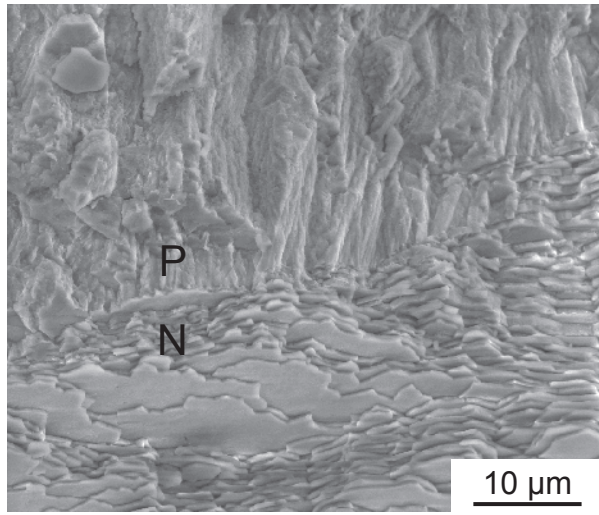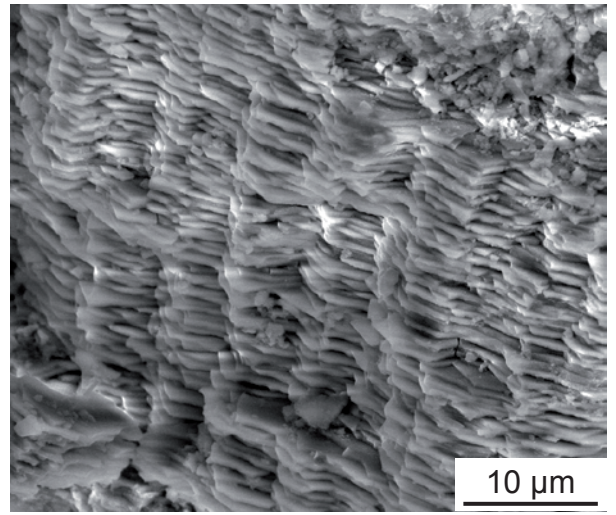

Supplement: Additional file 1 — Fractured shell at the nacre/prismatic transition Left image, Environmental scanning electron microscopy image of a fractured Haliotis pulcherina sea shell. The sharp transition from prismatic calcite (top layer) to aragonite nacre (bottom layer) is formed by differentiated shell forming regions of the mantle epithelial tissue. The biological regulation of such sharp transitions between different shell structures is still enigmatic. Right image, scanning electron microscopy image of the nacreous part of a fractured Haliotis pulcherina sea shell. The regular stacking of single nacre platelets is visualized, whereas a super-structure in the size range of up to 20 single platelets in vertical direction is not observed here. [file 2046-1682-5-19-S1.pdf]

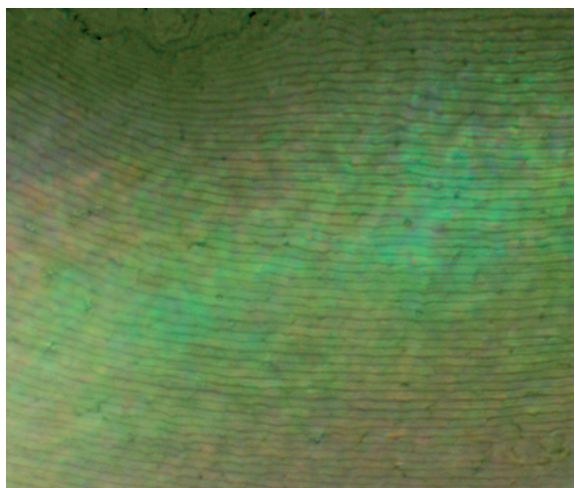

20  $\mu\text{m}$

Supplement: Additional file 2 — Incident light microscopy of nacre shell part. Incident light microscopy of polished shell cross-section in orientation A (compare also Figure 2C). In addition to the nacre lamellae there is another level of hierarchy in the range of ~20 μm, defined here as the super-structure, which is observed in orientation A at higher magnification. [file 2046-1682-5-19-S2.pdf]

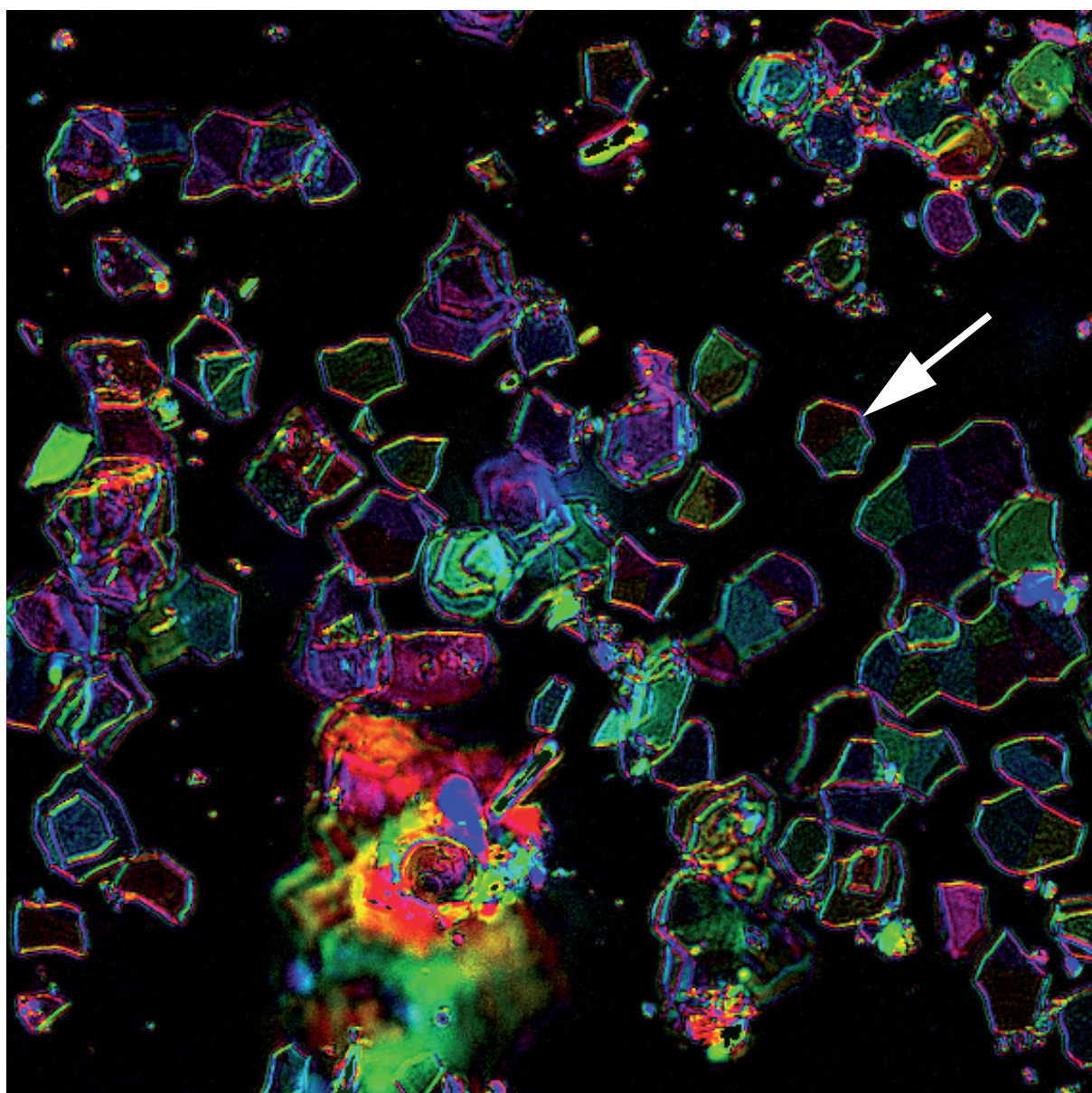

10  $\mu\text{m}$

Supplement: Additional file 4: — Birefringent retardance of isolated nacre platelets. LC-PolScope orientation image of isolated nacre tablets from Haliotis pulcherina sea shell. In addition to single nacre platelets (arrow), clusters of platelets attached to one another in a fragment of a single nacre layer are also visible. The overall birefringence in the c-axis direction of each platelet is weak, whereas the form birefringence at the boundary between mineral and water gives a bright contrast. This is demonstrated by the fact that confluent platelets show internal boundaries between single platelets with only weak retardance values. Dot-like structures are also visible, which have similar orientations within the imaging plane (a- and b-axis of the aragonite crystals) in certain domains of each platelet. The dot-like patterns could be internal or surface structures. If they are internal structures, they could be organic matrix shielded by the mineral phase or, replica voids as a result of sodium hypochlorite treatment, which was used to solubilize the interlamellar organic matrix. They could as well be the result of surface topography. [file 2046-1682-5-19-S4.pdf]
